# Early life injuries and the development of attention deficit/hyperactivity disorder

**DOI:** 10.4088/JCP.21m14033

**Published:** 2022-01-04

**Authors:** Theresa Wimberley, Isabell Brikell, Emil M Pedersen, Esben Agerbo, Bjarni J Vilhjálmsson, Clara Albiñana, Florian Privé, Anita Thapar, Kate Langley, Lucy Riglin, Marianne Simonsen, Helena S Nielsen, Anders D Børglum, Merete Nordentoft, Preben B Mortensen, Søren Dalsgaard

**Affiliations:** a iPSYCH - The Lundbeck Foundation Initiative for Integrative Psychiatric Research, Copenhagen and Aarhus, Denmark; b NCRR - National Centre for Register-based Research, Department of Economics and Business Economics, Aarhus University, Aarhus, Denmark; c CIRRAU - Centre for Integrated Register-based Research, Aarhus University, Aarhus, Denmark; d Division of Psychological Medicine and Clinical Neurosciences, MRC Centre for Neuropsychiatric Genetics and Genomics, Cardiff University, Cardiff, United Kingdom; e School of Psychology, Cardiff University, Cardiff, United Kingdom; f Department of Economics and Business Economics, School of Business and Social Sciences, Aarhus University, Aarhus, Denmark; g Department of Biomedicine and Centre for Integrative Sequencing, iSEQ, Aarhus University, Aarhus, Denmark; h Center for Genomics and Personalized Medicine, Central Region Denmark and Aarhus University, Aarhus, Denmark; i CORE, Mental Health Center Copenhagen, Copenhagen University Hospital, Denmark

**Keywords:** Attention deficit/hyperactivity disorder, injuries, familial aggregation, polygenic risk, genetic correlation

## Abstract

**Objectives:**

To estimate phenotypic and familial association between early-life injuries and attention deficit/hyperactivity disorder (ADHD) and the genetic contribution to the association using polygenic risk score for ADHD (PRS-ADHD) and genetic correlation analyses.

**Methods:**

Children born in Denmark 1995-2010 (n=786,543) were followed from age five until a median age of 14 (IQR: 10–18 years). Using ICD-10 diagnoses, we estimated hazard ratios (HRs) and absolute risks of ADHD by number of hospital/emergency ward treated injuries by age five. In a subsample born 1995 to 2005 with genetic data available (n=16,580), we estimated incidence rate ratios (IRR) for the association between PRS-ADHD and number of injuries before age five and the genetic correlation between ADHD and any injury before age five.

**Results:**

Injuries were associated with ADHD (HR=1.61; 95% CI 1.55-1.66), in males (HR=1.59; 1.53-1.65) and females (HR=1.65; 1.54-1.77), with a dose-response relationship with number of injuries. The absolute ADHD risk by age 15 was 8.4% (3+ injuries) vs. 3.1% (no injuries).

ADHD was also associated with injuries in relatives, stronger in 1^st^- than 2^nd^-degree relatives. PRS-ADHD was marginally associated with the number of injuries in the general population (IRR=1.06; 1.00-1.14), with a genetic correlation of 0.53 (0.21-0.85).

**Conclusions:**

Early-life injuries in individuals and their relatives were associated with a diagnosis of ADHD. However, even in children with the most injuries, more than 90% were not diagnosed with ADHD by age 15. Despite a low positive predictive value and that the impact of unmeasured factors such as parental behaviour remains unclear, results indicate that the association is partly explained by genetics, suggesting that early-life injuries may represent or herald early behavioral manifestations of ADHD.

## Introduction

Attention deficit/hyperactivity disorder (ADHD) is more difficult to validly diagnose before age five, as the diagnostic criteria relates mainly to older children, and the clinical presentation of ADHD may differ in pre-school and school. Besides subthreshold symptoms of inattention, hyperactivity, and impulsivity, little is known on early-life manifestations of ADHD.^1^ In typically developing children, impulsive and risk-taking behaviour is more common at a younger age,^2^ as are injuries.^3, 4^ The ability to plan ahead and foresee unsafe situations improves with age, as executive brain functions mature,^5^ and this cognitive maturation is paralleled by a decrease in the risk of injuries.^6^ Since the 1960s, studies of pre-school children have found injuries to be correlated with inattention, hyperactivity, and impulsivity.^7–11^ Meta-analyses provide strong evidence that individuals with ADHD have increased risk of emergency ward visits,^12^ and unintentional injuries.^13–15^ Traumatic brain injury (TBI) may be causally linked to ADHD,^16–18^ whereas literature on whether other injuries early in life tend to precede a diagnosis of ADHD is sparse.

ADHD is a neurodevelopmental disorder,^19^ with heritability estimates of around 74%.^20^ Independently derived ADHD polygenic risk score (ADHD-PRS) predicts ADHD-status,^21^ subthreshold ADHD-symptoms,^22^ and other neurodevelopmental traits in the general population.^23^ Genome-wide association studies (GWAS) recently identified several genome-wide significant loci, and estimated that common genetic variants explain 22% of the heritability of ADHD.^24^

Less is known about the aetiology of early-life injuries. Recently, a Danish register-based study found maternal depression to be associated with injuries during early childhood^25^ and depression and ADHD are genetically correlated.^24^ A twin study suggest a small genetic contribution to injuries before age five, with most variance explained by child-specific environmental effects (including ADHD-symptoms) and familial effects, several of which are known risk factors for ADHD, such as low socioeconomic status (SES), and young and single parenthood.^26^ Similarly, a Finnish twin study found little indication of a genetic contribution to injuries in adults.^27^ However, Acar et al. found that fathers of children admitted to the emergency ward due to an injury had higher ADHD symptom scores themselves, compared with fathers of children acutely admitted to hospital due to a throat infection.^28^ The cross-generational association between ADHD and injuries may be explained by home-environment, parental behaviour, parent-child interactions or genetics.

We hypothesize that similar to early subthreshold symptoms of ADHD, injuries before age five are early manifestations of ADHD and may associate with ADHD genetic liability. Using a large Danish population-based cohort, our aims were to (1) estimate the association between the number of injuries before age five and a later diagnosis of ADHD, (2) estimate the association between early-life injuries in siblings and parents and the risk of ADHD, (3) estimate the association between ADHD-PRS and the number of early-life injuries in the general population as well as the genetic correlations between ADHD and early-life injuries.

## Methods

### Data sources

Since 1968 the Danish Civil Registration System^29^ holds data on personal identification number, sex, date of birth, and continuously updated vital status of all persons living in Denmark, enabling accurate linkage of individual-level data across registers. The Danish National Patient Register (DNPR) and the Danish Psychiatric Central Research Register (DPCRR) holds data on all-inpatient admissions, and out-patient and emergency ward visits from 1995 onward and provided data on injuries and psychiatric disorders diagnosed in hospital departments.^30, 31^ Diagnoses were registered according to the International Classification of Diseases, 8^th^ revision (ICD-8) in 1969-93, and 10^th^ revision (ICD-10)^32^ since 1994. The Danish Neonatal Screening Biobank provided dried bloodspots and The Integrative Psychiatric Research (iPSYCH) consortium processed these for genotyping.^33^

### Study population

Population-based cohort study of individuals born in Denmark of Danish-born parents between January 1, 1995 and Dec 31, 2010, and living in Denmark at age five. Individuals were excluded if they fulfilled any of these criteria before age five: ADHD-diagnosis or filled prescriptions of ADHD-medication, TBI, or disease of the nervous system. Genetic analyses relied on the population-based iPSYCH2012 case-cohort, including all ADHD cases (N=18 726) and 2% random sample of the general population (sub-cohort) (N=30 000).^33^ DNA collection, genotyping and quality control has been described elsewhere.^33^ Included individuals fulfilled these criteria: Singletons, born between January 1, 1995 and December 31, 2005, alive and residing in Denmark at age 1, both parents born in Denmark and no diagnosis of TBI before age five. We further restricted to individuals of European ancestry^34^ and for genetic correlation analyses to unrelated individuals^35^. See Supplementary tables S1–S2 for codes and flow of exclusions of participants.

### Early-life injuries

We defined early-life injury in cohort members and their siblings as a hospital-treated injury before age five. This age cut-off ensured complete coverage of injuries and that injuries preceded the ADHD-diagnosis. We included diagnoses of injuries from all hospital contacts (inpatient, outpatient and emergency ward visits), excluding TBIs (Supplementary table 1) and contacts due to self-harm (ICD-10: X60-X84; or contact reason=4). These unintentional injuries, not including TBIs, are from now and onwards termed “injuries”. Injuries were defined as any (yes/no) and as number of injuries (0,1,2,3+). For number of injuries before age five, consecutive injury-contacts within 60 days were considered as one injury. An overview of injuries and their sex-distribution are shown in Supplementary table 1.

### Injuries in relatives

Parental injuries were based on ICD-8/10 codes (Supplementary table 1) and defined as at least two in-patient admissions due to injuries before 20 years of age, as data on outpatient contacts and emergency ward visits was only available from 1995 onwards. For sibling exposures, the study population was restricted to children having at least one full/half sibling, fulfilling the same inclusion criteria as the index child and exposure was defined as the number of injuries before age five. If the index child had more than one sibling, this was calculated as the average number of injuries before age five across the siblings, i.e. ≥1 vs. no injuries. For full siblings also in categories ]0,1], ]1,2], >2 vs. no injuries, in line with previous research.^36^

### Diagnoses of ADHD

Using data from DPCRR and DNPR, we defined ADHD as the first hospital contact to a psychiatric, paediatric or neurological department with a diagnosis of ADHD (ICD-10 codes F90.x, F98.8) after the age of five. ADHD subtypes included combined (F90.0) and inattentive subtype ADHD (F98.8). Based on funding available at the time, sampling of ADHD-cases for the iPSYHC2012 case-cohort sample only included cases with ICD-10 code F90.0.

### Polygenic risk scores for ADHD

PRS were derived using both LDpred^37^ and BOLT-LMM^38^ software and combined in a linear combination to obtain final PRS (see the Supplementary appendix 1).^39^ PRS-ADHD was standardized based on the mean and standard deviation in the iPSYCH sub-cohort, representing the distribution in the general population.

### Statistical analyses

First, hazard ratios (HRs) were estimated by Cox regression for the association between early-life injuries and ADHD, using age as the underlying time-axis. Analyses were repeated for the two ADHD subtypes and in strata of parental education (completed high-school yes/no). Individuals were followed from their 5^th^ birthday until first ADHD-diagnosis, TBI, emigration, or death, whichever came first. Absolute risks were calculated as cumulative incidences of ADHD at age 10 and 15 years.

Second, we investigated familial co-aggregation of injuries and ADHD, estimating HRs for associations between injuries in mothers, fathers, full siblings, maternal and paternal half-siblings (exposures), and ADHD in the index child (outcome). All analyses were adjusted for sex, birth cohort (1995-1998, 1999-2002, 2003-2006, 2007-2010), and the interaction between sex and birth cohort. Analyses of parental injuries were additionally adjusted for the parent’s birth cohort (<1968, 1968-1971, 1972-1977, >1977). Analyses of average number of injuries in siblings were additionally adjusted for the number of full/maternal half/paternal half siblings (1, 2, 3+).

Third, incidence rate ratios (IRRs) were estimated by negative binomial regression with the logarithm transformed time-at risk as an offset for associations between PRS-ADHD and the number of injuries before age five. Due to the iPSYCH2012 case-cohort sampling design, including all ADHD cases and a 2% random sub-cohort, inverse sampling probabilities were applied as weights.^40^ IRRs correspond to a relative increase in the rate of early-life injuries for a one standard deviation increase in the PRS-ADHD. These analyses were adjusted for sex, age and calendar year at first ADHD diagnosis (both continuous), genotyping wave (categorical), and the first four principal components (PCs) to adjust for potential remaining population stratification. The PCA method for deriving the PCs used for ancestry outlier removal and adjustment is described in detail elsewhere.^34^ Follow-up started at birth and ended at age five, a diagnosis of disease of the nervous system (ICD-10 codes G00-G99), death or emigration from Denmark, whichever came first.

All analyses were repeated in males and females and applied a cluster-robust variance estimator with clusters defined as individuals having the same mother and father and for half-sibling cohorts defined as individuals having the same mother or father.

Finally, SNP-heritability (h^2^SNP) and genetic correlation (r_g_) between early-life injuries and ADHD were estimated using BOLT-REML software among the iPSYCH2012 ADHD cases and subcohort. SNPs were filtered and LD pruned according to BOLT-REML suggested guidelines^35^ (for details, see Supplementary appendix 2). Heritability estimates were transformed to the liability scale as proposed by Lee et al.,^41^ assuming population prevalence of 5% for ADHD and 10% for injuries.

The main analyses were conducted using Stata 15.^42^ Plots of cumulative incidences were estimated and plotted using R 3.6.1.^43^ All estimates are accompanied by 95% confidence intervals (CIs).

### Ethics

iPSYCH is approved by the Danish Data Protection Agency, the Danish Health Data Authority, the Danish Scientific Ethics Committee, and the Danish Neonatal Screening Biobank Steering Committee.^33, 44, 45^

## Results

The study population consisted of 786 543 children born in Denmark between 1995 and 2010, followed for a total of 6 910 193 person-years, until a median age of 13.9 years (IQR: 9.9 – 18.0). In total, 92 691 (11.8%) individuals sustained an injury before age five and 23 107 (2.9%) individuals were diagnosed with ADHD after five years of age and during follow-up. In 34 452 (4.4%) study participants, follow-up ended due to death (n=423), emigration from Denmark (n=11 704), or TBI (including concussion) after age five (n=22 053).

### Association between injuries and ADHD

Having sustained at least one injury before age five was associated with a subsequent diagnosis of ADHD (HR=1.61; 95% CI 1.55-1.66). Furthermore, an increasing number of injuries was associated with an increased risk of ADHD, suggesting a dose-response relationship (Table 1). Children with three or more injuries had a 2.5-fold increased risk of ADHD (HR=2.48 95% CI 2.27-2.72), when compared to children with no injuries.

We found no significant interaction between injuries (yes/no) and sex (p = 0.455) and associations were similar in sex-specific analyses (Table 1).

We observed a dose-response relation with higher cumulative incidence of ADHD by increasing number of injuries before age five (Figure 1 and Supplementary table 3). By 15 years of age, the estimated risk of ADHD ranged from 3.05% (95% CI 3.00-3.10%) in children with no injuries to 8.43% (95% CI 7.64-9.22%) in children with three or more injuries. Similar patterns were seen in both sexes (higher absolute risks in males), for ADHD subtypes (strongest associations for the combined subtype), and across strata of parental education (Tables S4 and S5).

### Familial co-aggregation of injuries and ADHD

ADHD was associated with injuries in 1^st^-degree relatives, including mothers (HR=1.47; 1.32-1.64), fathers (HR=1.45; 1.33-1.57) and full siblings (HR=1.39; 1.33-1.46) (Figure 2 and Supplementary table 6). The association was somewhat weaker in 2^nd^-degree relatives, including maternal half siblings (HR=1.28; 1.18-1.40) and paternal half siblings (HR=1.18; 1.08-1.29). The number of injuries in full siblings also increased the HR of ADHD in the index child incrementally. An average number of >2 injuries in full siblings was associated with an increased risk of ADHD (HR=1.81; 1.54-2.14), when compared to the risk of ADHD in full siblings without injuries. Similar sex-specific trends were observed (Supplementary table 6).

### Genetics of ADHD and injuries

In a random sample of the general population (n=16 580), a higher PRS-ADHD was associated with a higher number of injuries before age five (IRR=1.06; 1.00-1.14), with similar-sized estimates were for males and females (Table 2). In unrelated individuals (n=14 333), we found moderate SNP-based heritability for ADHD and low SNP-based heritability for early life injuries, with strong evidence for genetic correlation between the two r_g_ = 0.53 (95% CI 0.21-0.85) (Table 3).

## Discussion

In this population-based cohort study of almost 800 000 children, early-life injuries were associated with a subsequent clinical diagnosis of ADHD. Having sustained at least one injury before age five resulted in a 64% higher risk of ADHD, relative to those without injuries before age five. The association showed a dose-response pattern, as increasing number of injuries was associated with incremental increased risks of ADHD. Children with three or more injuries had a 2.5-fold increased risk of subsequently being diagnosed with ADHD after age five, when compared with children without early-life injuries.

Decades of research provide strong evidence that ADHD is associated with a 39-53% increased risk of injuries,^15^ and that ADHD-medication reduces this risk.^13, 15^ ADHD is associated with more collisions when riding a bicycle, more risk-taking behaviour, impulsive decision-making,^46^ and in adults higher rates of serious traffic accidents.^47^ The risk of fatal injuries is also increased in individuals with ADHD and accidents is their most common cause of death.^48, 49^

Other than symptoms of inattention, hyperactivity and impulsivity, little is known about what characterises children with ADHD in their first years of life, prior to being diagnosed. Some retrospective studies have found that children attending emergency wards have higher rates of ADHD-symptoms, than other children.^7–11^ Here, we show for the first time using an objective measure, that early-life injury is strongly associated with later ADHD risk and that increasing number of injures before age five may be a marker of ADHD liability already prior to diagnosis.

We also found familial co-aggregation of injuries and ADHD, which suggests that the aetiology of the two phenotypes includes shared familial risks. The associations were stronger between 1^st^-degree than 2^nd^-degree relatives, and in the general population, genetic liability for ADHD, as indexed by ADHD polygenic risk scores, was marginally associated with early-life injuries. However, we also found a genetic correlation of 0.53 between ADHD and early-life injuries; a higher correlation than ADHD shows with other neuropsychiatric disorders.^24, 50, 51^ This suggests that their underlying genetic liability is partly shared. However, the SNP-based heritability of injuries was low (6%), in keeping with findings from previous twin studies that have found little evidence for a genetic contribution to injuries.^26, 27^ Other shared familial risks could be related the psychosocial environment in the family, which is associated with both injuries and ADHD (including e.g. family history of mental disorders,^52, 53^ parental unemployment,^54, 55^ and teenage parenthood.^26, 56^). Finally, our observations could also be explained by gene-environment correlation whereby genetic liability to ADHD increases the risk for environmental stressors; a phenomenon that to date has only been observed for social stressors.^57–59^

We find that the risk of injuries may be linked to individual and familial characteristics, and not merely caused by external factors, which supports a hypothesis suggested in 1919.^60^ Based on that, the concept of ‘accident proneness’ was presented in 1926.^61–63^ During the 1940s and 50s, several publications supported this concept, linking it with different psychiatric symptoms, including distractibility, impulsivity, aggression, impatience, opposition, restlessness, and hyperactivity,^64, 65^ and also documenting familial aggregation of accidents.^66, 67^ Later, the concept of ‘accident proneness’ was criticised for placing the responsibility of sustaining an injury solely on the injured individuals themselves.^68, 69^ While our data suggest shared liabilities for injuries and ADHD, and that individuals more prone to injuries are more likely to have ADHD, this still explains very little of the variance in early-life injuries.

Major strengths of our study include the longitudinally recorded individual-level data on a population-based national sample in a country with free universal health. Furthermore, we were able to link the individual information with information in parents and siblings and to link with genetic information on all with an ADHD diagnosis and randomly selected controls. The randomly selected cohort enables us to present valid estimates of absolute risk in the general population, something most studies cannot. Furthermore, our similar findings among individuals of high and low educated parents suggest that the association between injuries and ADHD is only minimally biased by differences in hospital-seeking behaviour due to SES. However, our study also has some important limitations. First, we did not estimate the potentially mediating or moderating effects of other mental disorders often comorbid to ADHD (e.g. conduct disorder). Second, with TBI excluded we did not hypothesize a direct causal relationship between early injuries and ADHD and hence, we decided not to adjust analyses for e.g. parental SES or mental disorders. However, the impact of home-environment, parental behaviour, or parent-child interactions was not assessed and hence we were not able to fully disentangle the genetic and non-genetic contributions to the observed association. Third, we relied on previously validated data from the national health registries on clinical diagnoses of ADHD from hospital departments, and persons diagnosed by child and adolescent psychiatrists in private practices were not included. According to Danish guidelines, inattention without hyperactivity should be coded as F98.8,^70^ yet the validity of this ICD-diagnosis in the Danish registers is not fully known.^71^ However, we expect this misclassification to be non-differential by groups of the injuries exposure, meaning our estimates of association might be underestimated. Fourth, while BOLT-REML seems to provide robust estimates for the genetic correlation in a setting with shared controls and case-control ascertainments, the estimated SNP-based heritabilities are likely underestimated and should be interpreted with caution.^72^ Finally, even in children with the most injuries, more than 90% were not diagnosed with ADHD. Hence, with such a low positive predictive value our data does not support obtaining information about early-life injuries should be recommended as part of the clinical assessment for ADHD, or that such information would improve diagnostic validity.

To conclude, our study adds important new findings to the literature on what characterizes children with ADHD, years before they are diagnosed and the data suggest that early-life injuries may be an early manifestation of impairment and risks related to symptoms of ADHD. Furthermore, the observed association between early-life injuries and ADHD is partly due to shared familial and genetic risks.

## Supplementary Material

Supplement

## Figures and Tables

**Figure 1:** Cumulative incidence functions for ADHD by number of injuries. note: Cumulative incidences of ADHD after age five with 95% confidence interval (y-axis) by age (x-axis), estimated for different exposure groups (≥3, 2, 1 and 0 injuries before age five). Curves are shown for the entire cohort (a) and for males (b) and females (c). Abbreviations: ADHD: attention deficit/hyperactivity disorder.

**Figure 2:** Associations between injuries and ADHD note: Individual and familial associations between injuries and ADHD in the index individual. Estimates are shown for the entire cohort and for males and females, separately. The exposure was defined as at least one injury before age five for individual and siblings and at least two inpatient admissions due to injuries before age 20 in parents. All analyses were adjusted for sex, birth cohort and the interaction between sex and birth cohort. Sibling exposure analyses were additionally adjusted for number of siblings. Parental exposure analyses were additionally adjusted for parent’s birth cohort. Abbreviations: ADHD: attention deficit/hyperactivity disorder.

**Table 1:** Rates and HRs of ADHD, comparing individuals with no vs. any injuries and vs. number of injuries before age five.

	N	person-years	N(ADHD)	HR (95% CI)
**All**	786 543	6 910 193	23 107	
No injuries	693 852	6 120 395	18 850	1
>=1 injury^*^	92 691	789 798	4 257	1.61 (1.55-1.66)
0	693 852	6 120 395	18 850	1
1	67 077	572 698	2 769	1.45 (1.40-1.51)
2	19 014	160 862	1 007	1.83 (1.72-1.95)
3+	6 600	56 239	481	2.48 (2.27-2.72)
**Males**	401 758	3 495 504	16 191	
No injuries	345 125	3 019 195	12 921	1
>=1 injury	56 660	476 309	3 270	1.59 (1.53-1.65)
0	345 125	3 019 195	12 921	1
1	39 936	336 618	2 108	1.45 (1.38-1.52)
2	12 219	101 929	770	1.75 (1.63-1.88)
3+	4 505	37 761	392	2.46 (2.22-2.72)
**Females**	384 758	3414689	6916	
No injuries	348 727	3101200	5929	1
>=1 injury	36 031	313489	987	1.65 (1.54-1.77)
0	348 727	3101200	5929	1
1	27 141	236080	661	1.46 (1.35-1.59)
2	6 795	58932	237	2.12 (1.86-2.41)
3+	2 095	18477	89	2.56 (2.07-3.16)

The proportional hazards assumption was checked by visual inspection of log-minus-log plots for the exposure variable (injuries (yes/no) and 0,1,2,3+ injuries) and adjustment variables (birth cohort and sex). HRs were adjusted for sex and birth cohort and the interaction between sex and birth cohort. Robust variance estimation was applied to account for siblings. ^*^Among all individuals (with or without ADHD) with an injury before age five, less than 1% of these had their first injury within the first year of life.

Abbreviations: HR: Hazard ratio, ADHD: attention deficit/hyperactivity disorder.

**Table 2:** Association between PRS-ADHD and number of injuries before five years of age in iPSYCH ADHD cases and sub-cohort (16 580) and in males (n=10 430) and females (n=6 150), separately.

Exposure – Cohort	Cases N	person-years	Total number of injuries before age five	Rates^a^ of injuries per 100 person-years	IRR^b,c^ for injuries (95% CI)
PRS – All	16 580	81 521	3 637	4.46 (4.32-4.61)	1.06 (1.00-1.14)
PRS – Males	10 430	51 258	2 686	5.24 (5.04-5.44)	1.04 (0.96-1.13)
PRS – Females	6 150	30 264	951	3.14 (2.95-3.35)	1.09 (0.98-1.22)

a Rates are here presented unadjusted and unweighted.

b Analyses were adjusted for sex, genotyping wave, the first four principal components to correct for population stratification, and birth cohort. Cluster-robust variance estimation were applied to account for clustering by siblings.

c Adjusted and weighted IRR were estimated from a negative binomial regression analysis and weighted to represent associations in the general population.

Abbreviations: ADHD: attention deficit/hyperactivity disorder, iPSYCH: The Lundbeck Foundation Initiative for Integrative Psychiatric Research, IRR: Incidence rate ratio, PRS: Polygenic risk score.

**Table 3:** SNP-based heritability estimates of ADHD and early-life injuries and genetic correlation calculated^a^ in iPSYCH ADHD cases and sub-cohort (n =14 333).

Phenotype	Cases N	SNP-based heritability h^2^_SNP_ (95% CI)	Liability scale heritability^b^ h^2^_liab_ (95% CI)	Genetic correlation r_g_ (95% CI)
ADHD	6 186 (43.2%)	0.33 (0.28-0.39)	0.28 (0.24-0.33)	0.53 (0.21-0.85)
Any injury before age five	2 137 (14.9%)	0.06 (0.01-0.11)	0.13 (0.02-0.23)	

a BOLT-REML estimation on an LD-pruned set of SNPs (n=785 388).

b Heritability estimates were transformed to the liability scale assuming population prevalence of 5% for ADHD and 10% for injuries.. Sample prevalence were higher (43% and 16%, respectively) mainly due to oversampling of ADHD cases in the iPSYCH sample.

Abbreviations: ADHD: attention deficit/hyperactivity disorder, h^2^_SNP_: SNP-based heritability, h^2^_liab_: Liability scale heritability, iPSYCH: The Lundbeck Foundation Initiative for Integrative Psychiatric Research, LD: Linkage disequlibrium, REML: Restricted maximum likelihood, r_g_: Genetic correlation, SNP: single nucleotide polymorphism.

## Clinical Points

1. ADHD associates with increased injury-risk. Yet, it is unknown whether injuries in preschool are early manifestations of ADHD and possibly linked to ADHD-genetics in the general population.
2. In clinical assessments of school-children suspected of having ADHD, information on number of sustained injuries in preschool may further support the diagnosis of ADHD, as these behaviours are genetically linked to one-another.

## References

[1] Sonuga-Barke EJ, Daley D, Thompson M, Swanson J (2003). Preschool ADHD: exploring uncertainties in diagnostic validity and utility, and treatment efficacy and safety. Expert Rev Neurother.

[2] Dal Santo JA, Goodman RM, Glik D, Jackson K (2004). Childhood unintentional injuries: factors predicting injury risk among preschoolers. J Pediatr Psychol.

[3] Bourguet CC, McArtor RE (1989). Unintentional injuries. Risk factors in preschool children. Am J Dis Child.

[4] Ribeiro MGC, Paula ABR, Bezerra MAR, Rocha SSD, Avelino F, Gouveia MTO (2019). Social determinants of health associated with childhood accidents at home: An integrative review. Rev Bras Enferm.

[5] Seidman LJ, Biederman J, Monuteaux MC, Valera E, Doyle AE, Faraone SV (2005). Impact of gender and age on executive functioning: do girls and boys with and without attention deficit hyperactivity disorder differ neuropsychologically in preteen and teenage years?. DevNeuropsychol.

[6] Garzon DL (2005). Contributing factors to preschool unintentional injury. J Pediatr Nurs.

[7] Klein D (1969). Some applications of delinquency theory to childhood accidents. Pediatrics.

[8] Matheny AP (1986). Injuries Among Toddlers: Contributions from Child, Mother, and Family. Journal of Pediatric Psychology.

[9] Langley J, McGee R, Silva P, Williams S (1983). Child behavior and accidents. J Pediatr Psychol.

[10] Speltz ML, Gonzales N, Sulzbacher S, Quan L (1990). Assessment of injury risk in young children: a preliminary study of the injury behavior checklist. J Pediatr Psychol.

[11] Piazza-Waggoner C, Dotson C, Adams CD, Joseph K, Goldfarb IW, Slater H (2005). Preinjury Behavioral and Emotional Problems Among Pediatric Burn Patients. Journal of Burn Care and Rehabilitation.

[12] Dalsgaard S, Nielsen HS, Simonsen M (2014). Consequences of ADHD medication use for children’s outcomes. Journal of Health Economics.

[13] Dalsgaard S, Leckman JF, Mortensen PB, Nielsen HS, Simonsen M (2015). Effect of drugs on the risk of injuries in children with attention deficit hyperactivity disorder: a prospective cohort study. Lancet Psychiatry.

[14] Lindemann C, Langner I, Banaschewski T, Garbe E, Mikolajczyk RT (2017). The Risk of Hospitalizations with Injury Diagnoses in a Matched Cohort of Children and Adolescents with and without Attention Deficit/Hyperactivity Disorder in Germany: A Database Study. Front Pediatr.

[15] Ruiz-Goikoetxea M, Cortese S, Aznarez-Sanado M (2018). Risk of unintentional injuries in children and adolescents with ADHD and the impact of ADHD medications: A systematic review and metaanalysis. Neurosci Biobehav Rev.

[16] Adeyemo BO, Biederman J, Zafonte R (2014). Mild traumatic brain injury and ADHD: a systematic review of the literature and meta-analysis. Journal of attention disorders.

[17] Chang HK, Hsu JW, Wu JC (2018). Traumatic Brain Injury in Early Childhood and Risk of Attention-Deficit/Hyperactivity Disorder and Autism Spectrum Disorder: A Nationwide Longitudinal Study. J Clin Psychiatry.

[18] Stojanovski S, Felsky D, Viviano JD (2019). Polygenic Risk and Neural Substrates of Attention-Deficit/Hyperactivity Disorder Symptoms in Youths With a History of Mild Traumatic Brain Injury. Biol Psychiatry.

[19] American Psychiatric Association (2013). Diagnostic and Statistical Manual of Mental Disorders (5th edition).

[20] Faraone SV, Larsson H (2019). Genetics of attention deficit hyperactivity disorder. Mol Psychiatry.

[21] Stergiakouli E, Martin J, Hamshere ML (2015). Shared genetic influences between attention-deficit/hyperactivity disorder (ADHD) traits in children and clinical ADHD. Journal of the American Academy of Child and Adolescent Psychiatry.

[22] Riglin L, Collishaw S, Thapar AK (2016). Association of Genetic Risk Variants With Attention-Deficit/Hyperactivity Disorder Trajectories in the General Population. JAMA Psychiatry.

[23] Martin J, Hamshere ML, Stergiakouli E, O’Donovan MC, Thapar A (2014). Genetic risk for attention-deficit/hyperactivity disorder contributes to neurodevelopmental traits in the general population. Biological psychiatry.

[24] Demontis D, Walters RK, Martin J (2019). Discovery of the first genome-wide significant risk loci for attention deficit/hyperactivity disorder. Nat Genet.

[25] Lyngsoe BK, Munk-Olsen T, Vestergaard CH, Rytter D, Christensen KS, Bech BH (2021). Maternal depression and childhood injury risk: A population-based cohort study in Denmark. Brain Behav.

[26] Ordonana JR, Caspi A, Moffitt TE (2008). Unintentional injuries in a twin study of preschool children: environmental, not genetic, risk factors. J Pediatr Psychol.

[27] Salminen S, Vuoksimaa E, Rose RJ, Kaprio J (2018). Age, Sex, and Genetic and Environmental Effects on Unintentional Injuries in Young and Adult Twins. Twin Res Hum Genet.

[28] Acar E, Dursun OB, Esin IS, Ogutlu H, Ozcan H, Mutlu M (2015). Unintentional Injuries in Preschool Age Children: Is There a Correlation With Parenting Style and Parental Attention Deficit and Hyperactivity Symptoms. Medicine (Baltimore).

[29] Pedersen CB, Gøtzsche H, Møller JØ, Mortensen PB (2006). The Danish Civil Registration System - A cohort of eight million persons. Danish medical bulletin.

[30] Schmidt M, Schmidt SA, Sandegaard JL, Ehrenstein V, Pedersen L, Sorensen HT (2015). The Danish National Patient Registry: a review of content, data quality, and research potential. Clin Epidemiol.

[31] Mors O, Perto GP, Mortensen PB (2011). The Danish Psychiatric Central Research Register. ScandJPublic Health.

[32] World Health Organization (1992). The ICD-10 Classification of Mental and Behavioural Disorders: Clinical descriptions and diagnostic guidelines.

[33] Pedersen CB, Bybjerg-Grauholm J, Pedersen MG (2018). The iPSYCH2012 case-cohort sample: new directions for unravelling genetic and environmental architectures of severe mental disorders. Mol Psychiatry.

[34] Prive F, Luu K, Blum MGB, McGrath JJ, Vilhjalmsson BJ (2020). Efficient toolkit implementing best practices for principal component analysis of population genetic data. Bioinformatics.

[35] Loh PR, Bhatia G, Gusev A (2015). Contrasting genetic architectures of schizophrenia and other complex diseases using fast variance-components analysis. Nat Genet.

[36] Debost JC, Larsen JT, Munk-Olsen T, Mortensen PB, Agerbo E, Petersen LV (2019). Childhood infections and schizophrenia: The impact of parental SES and mental illness, and childhood adversities. Brain Behav Immun.

[37] Vilhjalmsson BJ, Yang J, Finucane HK (2015). Modeling Linkage Disequilibrium Increases Accuracy of Polygenic Risk Scores. Am J Hum Genet.

[38] Loh PR, Tucker G, Bulik-Sullivan BK (2015). Efficient Bayesian mixed-model analysis increases association power in large cohorts. Nat Genet.

[39] Albinana C, Grove J, McGrath JJ (2021). Leveraging both individual-level genetic data and GWAS summary statistics increases polygenic prediction. Am J Hum Genet.

[40] Borgan O, Langholz B, Samuelsen SO, Goldstein L, Pogoda J (2000). Exposure stratified case-cohort designs. Lifetime Data Anal.

[41] Lee SH, Goddard ME, Wray NR, Visscher PM (2012). A better coefficient of determination for genetic profile analysis. Genet Epidemiol.

[42] Stata (2016). Release 14 Statistical Software.

[43] R Development Core Team (2010). R: A language and environment for statistical programming.

[44] Mortensen PB (2019). Response to “Ethical concerns regarding Danish genetic research”. Mol Psychiatry.

[45] Norgaard-Pedersen B, Hougaard DM (2007). Storage policies and use of the Danish Newborn Screening Biobank. J Inherit Metab Dis.

[46] Nikolas MA, Elmore AL, Franzen L, O’Neal E, Kearney JK, Plumert JM (2016). Risky bicycling behavior among youth with and without attention-deficit hyperactivity disorder. Journal of child psychology and psychiatry, and allied disciplines.

[47] Chang Z, Lichtenstein P, D’Onofrio BM, Sjolander A, Larsson H (2014). Serious transport accidents in adults with attention-deficit/hyperactivity disorder and the effect of medication: a populationbased study. JAMA Psychiatry.

[48] Dalsgaard S, Ostergaard SD, Leckman JF, Mortensen PB, Pedersen MG (2015). Mortality in children, adolescents, and adults with attention deficit hyperactivity disorder: a nationwide cohort study. Lancet.

[49] Sun S, Kuja-Halkola R, Faraone SV (2019). Association of Psychiatric Comorbidity With the Risk of Premature Death Among Children and Adults With Attention-Deficit/Hyperactivity Disorder. JAMA Psychiatry.

[50] Anttila V, Bulik-Sullivan B, The Brainstorm Consortium (2018). Analysis of shared heritability in common disorders of the brain. Science.

[51] Cross-Disorder Group of the Psychiatric Genomics Consortium (2019). Genomic Relationships, Novel Loci, and Pleiotropic Mechanisms across Eight Psychiatric Disorders. Cell.

[52] Nevriana A, Pierce M, Dalman C (2020). Association between maternal and paternal mental illness and risk of injuries in children and adolescents: nationwide register based cohort study in Sweden. BMJ.

[53] McCoy BM, Rickert ME, Class QA, Larsson H, Lichtenstein P, D’Onofrio BM (2014). Mediators of the association between parental severe mental illness and offspring neurodevelopmental problems. Ann Epidemiol.

[54] Sideri S, Marcenes W, Stansfeld SA, Bernabe E (2018). Family environment and traumatic dental injuries in adolescents. Dent Traumatol.

[55] Keilow M, Wu C, Obel C (2020). Cumulative social disadvantage and risk of attention deficit hyperactivity disorder: Results from a nationwide cohort study. SSM Popul Health.

[56] Ostergaard SD, Dalsgaard S, Faraone SV, Munk-Olsen T, Laursen TM (2017). Teenage Parenthood and Birth Rates for Individuals With and Without Attention-Deficit/Hyperactivity Disorder: A Nationwide Cohort Study. Journal of the American Academy of Child and Adolescent Psychiatry.

[57] Harold GT, Leve LD, Barrett D (2013). Biological and rearing mother influences on child ADHD symptoms: revisiting the developmental interface between nature and nurture. J Child Psychol Psychiatry.

[58] Stern A, Agnew-Blais J, Danese A (2018). Associations between abuse/neglect and ADHD from childhood to young adulthood: A prospective nationally-representative twin study. Child Abuse Negl.

[59] Ostergaard SD, Trabjerg BB, Als TD (2020). Polygenic risk score, psychosocial environment and the risk of attention-deficit/hyperactivity disorder. Transl Psychiatry.

[60] Greenwood M, Woods HM (1919). The incidence of industrial accidents upon individuals, with special reference to multiple accidents.

[61] Farmer E, Chambers EG (1926). A Psychological Study of Individual Differences in Accident Rates.

[62] Marbe K (1926). Praktische Psychologie der Unfälle und Betriebsschäden.

[63] Newbold EM (1926). A Contribution to the Study of the Human Factor in the Causation of Accidents.

[64] Bakwin RM, Bakwin H (1948). Accident proneness. The Journal of Pediatrics.

[65] Dalsgaard-Nielsen T, Dirksen H (1954). HEAD INJURY AND ACCIDENT-PRONENESS (SOMATO-PSYCHIC CAUSES OF SUCH INJURIES). Acta psychiatrica Scandinavica.

[66] Partington MW (1960). The importance of accident-proneness in the aetiology of head injuries in childhood. Arch Dis Child.

[67] Dunbar F (1943). Psychosomatic Diagnosis.

[68] Froggatt P, Smiley JA (1964). The Concept of Accident Proneness: A Review. Br J Ind Med.

[69] McKenna FP (1983). Accident proneness: A conceptual analysis. Accident Analysis & Prevention.

[70] Sundhedsstyrrelsen. [Danish Health Authority] (2018). National klinisk retningslinje for udredning og behandling af ADHD hos børn og unge [National clinical guideline for assessment and treatment of ADHD in children and adolescents].

[71] Mohr-Jensen C, Vinkel Koch S, Briciet Lauritsen M, Steinhausen HC (2016). The validity and reliability of the diagnosis of hyperkinetic disorders in the Danish Psychiatric Central Research Registry. Eur Psychiatry.

[72] Weissbrod O, Flint J, Rosset S (2018). Estimating SNP-Based Heritability and Genetic Correlation in Case-Control Studies Directly and with Summary Statistics. Am J Hum Genet.

