# Supplementary material for: Early life injuries and the development of attention deficit/hyperactivity disorder
Source: J Clin Psychiatry. Author manuscript; Available in PMC 2022 Feb 4. (PMC7612325; doi:10.4088/JCP.21m14033)
Supplement: Supplement [file EMS140872-supplement-Supplement.docx]

**SUPPLEMENTARY MATERIAL**

# Appendix 1. Calculation of polygenic risk scores for ADHD (PRS-ADHD)

The polygenic risk score for ADHD (PRS-ADHD) was calculated using both internal (to iPSYCH2012) and external SNP weights (from external GWAS summary statistics). We derived externally trained PRS-ADHD using the LDpred software, specifying an infinitesimal model.^1^ SNP weights were obtained from publicly available external ADHD GWAS summary statistics (1947 trio cases and pseudo-controls, 840 case and 688 controls),^2^ selecting European ancestry discovery GWAS without the iPSYCH2012 sample included. The LDPred PRS was derived for a set of genotyped SNPs (n=166 329, filtered for minor allele frequency (MAF) > 1% and missing values < 10%) overlapping between the iPSYCH2012 sample and the external GWAS summary statistics and restricted to HapMap3 variants (v1.2).

Owing to the large number of ADHD cases in iPSYCH2012, we also derived another set of internally trained PRS-ADHD in an unrelated ($\hat{\pi}$ coefficient > 0.2 using PLINK--rel-cutoff), European ancestry subset of the iPSYCH2012 sample. The internally trained SNP weights were obtained using the BOLT-LMM software.^3^ We performed a mixed model prediction of ADHD (i.e. best linear unbiased prediction [BLUP]) in which genotyped SNPs in the iPSYCH sample (n = 544 758, filtered for minor allele frequency > 0.01 and SNP missing rate < 0.1) were included as random effects. Prediction effect sizes from this model take into account LD between nearby SNPs to correctly weigh their contribution to the phenotypic variance (see supplementary material of Loh et al, 2015).^3^ To avoid overfitting, we used 10-fold cross-validation, training the model using 9/10ths of the data and testing it in the remaining tenth. The internally trained PRS was defined as the weighted sum of the training set prediction betas on the test set genotypes. The model was adjusted for genotyping wave, sex, age, and the first 10 principal components (PCs). The final PRS-ADHD was a linear combination of the internally and externally trained PRS variables, where the regression coefficients were inferred using two-fold cross validation on the test set. These PRS were standardized to the mean and standard deviation of the iPSYCH2012 control population.^4^

PRS were derived at the secured national GenomeDK high-performance computing cluster in Denmark and then imported to Statistics Denmark secure servers for linkage to other registry information. Further details on this procedure for PRS calculation can be found in a recently published paper.^5^

# Appendix 2. Calculation of SNP-heritability and genetic correlation

SNP-heritability (h^2^_SNP_) and genetic correlation (r_g_) between early-life injuries and ADHD were estimated using BOLT-REML software^6^ among the iPSYCH2012 ADHD cases and subcohort. The sample was restricted to individuals of European ancestry based on PCA analysis.^7^ Furthermore, individuals were filtered for relatedness using PLINK command --rel-cutoff 0.05.^6^ Genotyped and imputed SNPs were filtered for MAF > 1%, SNP missing rate < 5% and info score > 0.8 according to BOLT-REML suggested guidelines^6^. SNPs were then LD-pruned in two rounds in PLINK version1.90^8-10^ using command --indep-pairwise (50 5 0.9), resulting in a number of 785 388 SNPs used for analysis. Finally, heritability estimates were transformed to the liability scale as proposed by Lee et al.,^11^ assuming population prevalence of 5% for ADHD and 10% for injuries.

Table 1 ICD-10 codes and frequencies of types of injuries before age five *in boys and girls in the study population*

| **Type of injury** | **ICD-10 codes** | **Individuals with injury before age five, N (%)^a^** | | |
| --- | --- | --- | --- | --- |
|  |  | All  (n=786 543) | Males  (n= 401 785) | Females  (n=384 758) |
| Traumatic brain injury (TBI)^b,c^ | S02.0-1, S02.3, S02.7-9, S06, S07, S09.0, S09.7-9, S18,  T02.0, T04.0, T06.0, T90.2, T90.5, T90.8-9 |  |  |  |
| Any non-TBI injury^d,e^ | Any of the codes below | 92 691 (11.8) | 56 660 (14.1) | 36 031 (9.4) |
| Severe and multiple injuries and traumatic amputation | S28, S38, S48, S58, S68.3, S68.4, S68.8, S68.9, S78, S88, S98.0, S98.3, S98.4, T05, T09.6, T11.6, T13.6, T14.7 | 10 (0.01) | 5 (0.0) | 5 (0.0) |
| Fracture | S02.2, S02.4-6, S12, S17, S22, S32, S42, S47, S52, S57, S62, S67, S68.0, S68.1, S68.2, S72, S77, S82, S87, S92,  S97, S98.1, S98.2, T02, T04, T08, T10, T12, T14.2 | 13 479 (1.7) | 8 030 (2.0) | 5 449 (1.4) |
| Internal organs | S26, S27, S36, S37 | 48 (0.0) | 34 (0.0) | 14 (0.0) |
| Open wounds^f^ | S01, S04, S05, S08, S11, S14-S16, S18, S19, S21, S24, S25, S29, S31, S34, S35, S39, S41, S44-S46, S49, S51, S54-S56, S59, S61 S64-S66, S69, S71, S74-S76, S79, S81, S84-S86, S89, S91, S94-S96, S99, T01, T06, T07, T09.1, T09.3-5, T09.8, T09.9, T11.1, T11.3-5, T11.8, T11.9, T13.1, T13.3-5, T13.8, T13.9, T14.1, T14.4, T14.5, T14.8, T14.9 | 44 393 (5.6) | 29 727 (7.4) | 14 666 (3.8) |
| Dislocation, sprain and strain | S23, S33, S43, S53, S63, S73, S83, S93, T03, T09.2, T11.2, T13.2, T14.3 | 11 239 (1.4) | 5 560 (1.4) | 5 679 (1.5) |
| Superficial injury | S00, S10, S20, S30, S40, S50, S60, S70, S80, S90, T00, T09.0, T11.0, 13.0, T14.0 | 33 902 (4.3) | 20 902 (5.2) | 13 000 (3.4) |
| Burns | T20-T31 | 2 964 (0.4) | 1 793 (0.4) | 1 171 (0.3) |
| Foreign bodies | T15-19 | 7 724 (1.0) | 4 190 (1.0) | 3 534 (0.9) |

^a^ Prevalence of types of injuries do not sum to the prevalence of any injury, as individuals may have been diagnosed with several types of injuries before age five.

^b^ Individuals with TBI before age five (n=22 337, 12 136 males and 9 917 females) were not included in the final study population.

^c^ In parents, ICD-8 codes N800-804, 850-854 were additionally used to identify TBI.

^d^ To include only unintentional injuries, any hospital contacts due to self-harm were excluded (ICD-10 code X60-X84 or reason for contact code 4). In parents, ICD-8 codes E950-959 were additionally used to identify self-harm.

^e^ In parents, ICD-8 codes N805-849, 855-859 were additionally used to identify non-TBI injuries.

^f^ Other and unspecified injuries are included in the category ‘open wounds’.

Abbreviations: ICD-8: International classification of diseases, 8^th^ revision, ICD-10: International Classification of Diseases, 10^th^ revision, TBI: traumatic brain injury.

#

# Table 2 Inclusion criteria for study populations I-VI

| **Inclusion criteria** |  | **Excluded, N(%)** | **Included, N** |
| --- | --- | --- | --- |
| Study population I: General population | | | |
| All born Jan 1, 1995 - Dec 31, 2010 |  |  | 1 191 976 |
| Born in Denmark |  | 144 346 (12.1) | 1 047 630 |
| Alive at age five |  | 3 931 (0.4) | 1 043 688 |
| Not emigrated at age five |  | 31 530 (3.0) | 1 012 169 |
| No diagnosis of ADHD before age five |  | 1 948 (0.2) | 1 010 221 |
| No prescriptions of ADHD medication before age five |  | 35 (0.0) | 1 010 186 |
| No diagnosis of disease of the nervous system (ICD-10: G00-G99) before age five |  | 15 812 (1.6) | 994 374 |
| Mother born in Denmark |  | 132 229 (13.3) | 862 145 |
| Farther born in Denmark |  | 53 549 (6.2) | 808 596 |
| No TBI before age five |  | 22 053 (2.7) | 786 543 |
| Study population II: Full siblings | | | |
| At least one full sibling and not included in the adoption register |  |  | 490 472 |
| Study population III: Maternal half siblings | | | |
| At least one maternal half sibling and not included in the adoption register |  |  | 57 689 |
| Study population IV: Paternal half siblings | | | |
| At least one paternal half sibling and not included in the adoption register |  |  | 54 602 |
| Study population V ^a^: iPSYCH data for PRS analyses | | | |
| ADHD cases and subcohort, not mutually exclusive |  |  | 48 339 |
| Genetic information, including PRS-ADHD |  | 5 300 (11.0) | 43 039 |
| All born May 1, 1995 - Dec 31, 2005 |  | 21 904 (50.9) | 21 135 |
| Mother born in DK |  | 2 285 (10.8) | 18 850 |
| Father born in DK |  | 1 030 (5.5) | 17 820 |
| No TBI before age five |  | 545 (3.1) | 17 107 |
| European ancestry |  | 527 (3.1) | 16 580 |
| Study population VI: iPSYCH data for genetic correlations | | | |
| Similar inclusion criteria as for study population V |  |  | 16 580 |
| Unrelated individuals identified by a relatedness threshold of 0.05 as recommended for the BOLT-REML procedure.^6^ |  | 2 247 (13.6) | 14 333 |

Abbreviations: ADHD: attention deficit hyperactivity disorder, ICD-10: International Classification of Diseases, 10^th^ revision,

iPSYCH: The Lundbeck Foundation Initiative for Integrative Psychiatric Research, PRS: Polygenic risk score, REML: Restricted maximum likelihood, TBI: traumatic brain injury.

Table 3 Number of ADHD cases and absolute risks of ADHD by age 10 and 15 years, *calculated for the entire cohort (n=22 794) and for males (n=15 994) and females (n=6 800), separately*

|  | **Number of incident ADHD cases** | | **Risk of ADHD (%)** | |
| --- | --- | --- | --- | --- |
| **Number of injuries** | **By age**  **10 years** | **By age**  **15 years** | **By age**  **10 years** | **By age**  **15 years** |
| All | 11 818 | 22 264 | 1.72 (1.69-1.75) | 3.33 (3.28-3.37) |
| 0 | 9 475 | 18 112 | 1.56 (1.53-1.59) | 3.05 (3.00-3.10) |
| 1 | 1 507 | 2 698 | 2.57 (2.45-2.70) | 4.87 (4.68-5.07) |
| 2 | 557 | 986 | 3.36 (3.09-3.64) | 6.31 (5.89-6.73) |
| 3+ | 279 | 468 | 4.83 (4.27-5.38) | 8.43 (7.64-9.22) |
| Males | 9 246 | 15 833 | 2.63 (2.58-2.69) | 4.79 (4.71-4.87) |
| 0 | 7 320 | 12 620 | 2.43 (2.37-2.48) | 4.43 (4.35-4.51) |
| 1 | 1 227 | 2 072 | 3.53 (3.33-3.72) | 6.46 (6.17-6.75) |
| 2 | 462 | 759 | 4.33 (3.94-4.72) | 7.53 (6.97-8.09) |
| 3+ | 237 | 382 | 6.04 (5.29-6.79) | 10.35 (9.30-11.40) |
| Females | 2 572 | 6 431 | 0.76 (0.73-0.79) | 1.81 (1.75-1.86) |
| 0 | 2 155 | 5 492 | 0.71 (0.68-0.74) | 1.69 (1.64-1.74) |
| 1 | 280 | 626 | 1.18 (1.04-1.32) | 2.56 (2.33-2.80) |
| 2 | 95 | 227 | 1.62 (1.29-1.94) | 4.11 (3.51-4.70) |
| 3+ | 42 | 86 | 2.25 (1.58-2.93) | 4.40 (3.36-5.45) |

Abbreviations: ADHD: attention deficit hyperactivity disorder.

*Table 4 Hazard ratios of different subtypes of ADHD*, comparing individuals with no vs. any injuries and vs. number of injuries before age five

| **ADHD subtypes** | **All** | | **Males** | | **Females** | |
| --- | --- | --- | --- | --- | --- | --- |
|  | **ADHD cases, N** | **HR^a^ (95% CI)** | **ADHD cases, N** | **HR^a^ (95% CI)** | **ADHD cases, N** | **HR^a^ (95% CI)** |
| Combined subtype (F90.0) | 15688 |  | 11407 |  | 4281 |  |
| No injuries | 12718 | 1 | 9055 | 1 | 3663 | 1 |
| >=1 injury | 2970 | 1.64 (1.57-1.71) | 2352 | 1.63 (1.56-1.70) | 618 | 1.67 (1.53-1.82) |
| Inattentive subtype (F98.8) | 4521 |  | 2719 |  | 1802 |  |
| No injuries | 3800 | 1 | 2238 | 1 | 1562 | 1 |
| >=1 injury | 721 | 1.40 (1.29-1.52) | 481 | 1.34 (1.22-1.48) | 240 | 1.51 (1.32-1.73) |

# Abbreviations: ADHD: attention deficit hyperactivity disorder, HR: Hazard ratio.

#

# Table 5 Association between injuries and ADHD across parental education level

| **Parental education level^a^** | **All** | | **Males** | | **Females** | |
| --- | --- | --- | --- | --- | --- | --- |
|  | **ADHD cases, N** | **HR^a^ (95% CI)** | **ADHD cases, N** | **HR^a^ (95% CI)** | **ADHD cases, N** | **HR^a^ (95% CI)** |
| Maternal low (n=145 728) | 8 513 |  | 5 832 |  | 2 681 |  |
| No injuries | 6 839 | 1 | 4 564 | 1 | 2 275 |  |
| >=1 injury | 1 674 | 1.48 (1.41-1.57) | 1 268 | 1.48 (1.39-1.58) | 406 | 1.49 (1.34-1.66) |
| Maternal higher (n=639 172) | 14 474 |  | 10 277 |  | 4 197 |  |
| No injuries | 11 917 | 1 | 8 295 | 1 | 3 622 |  |
| >=1 injury | 2 557 | 1.59 (1.52-1.66) | 1 982 | 1.57 (1.49-1.64) | 575 | 1.65 (1.51-1.80 |
| Paternal low (n=154 330) | 8 461 |  | 5 822 |  | 2 639 |  |
| No injuries | 6 822 | 1 | 4 586 | 1 | 2 242 |  |
| >=1 injury | 1 639 | 1.54 (1.46-1.63) | 1 242 | 1.53 (1.44-1.63) | 397 | 1.57 (1.41-1.75) |
| Paternal higher (n=627 888) | 14 286 |  | 10 132 |  | 4 154 |  |
| No injuries | 11 738 | 1 | 8 155 | 1 | 3 583 |  |
| >=1 injury | 2 548 | 1.59 (1.52-1.66) | 1 977 | 1.57 (1.50-1.65) | 571 | 1.63 (1.49-1.78) |

^a^ Individuals with missing information on maternal (n=1 643, 0.2%) or paternal (n=4 325, 0.6%) education level were not included in the respective analysis.

# Abbreviations: ADHD: attention deficit hyperactivity disorder, HR: Hazard ratio.

Table 6 Familial aggregation of injuries and ADHD*, with the association between parents and siblings (average) exposure to injuries and occurrence of ADHD in the index individual, estimated as*

*hazard ratios (HR) with 95% CI*

|  | **All** | | **Males** | | **Females** | |
| --- | --- | --- | --- | --- | --- | --- |
| **Injury exposures** | **ADHD cases, N** | **HR^a^ (95% CI)** | **ADHD cases, N** | **HR^a^ (95% CI)** | **ADHD cases, N** | **HR^a^ (95% CI)** |
| Within individual (index child) | 23 107 |  | 16 191 |  | 6916 |  |
| No injuries | 18 850 | 1 | 12 921 | 1 | 5929 | 1 |
| >=1 injury | 4 257 | 1.61 (1.55-1.66) | 3 270 | 1.59 (1.53-1.65) | 987 | 1.65 (1.54-1.77) |
| Mothers | 23107 |  | 16191 |  | 6916 |  |
| <2 injuries | 22706 | 1 | 15922 | 1 | 6784 | 1 |
| 2+ injuries | 401 | 1.47 (1.32-1.64) | 269 | 1.40 (1.24-1.59) | 132 | 1.65 (1.38-1.97) |
| Fathers | 23107 |  | 16191 |  | 6916 |  |
| <2 injuries | 22467 | 1 | 15747 | 1 | 6720 | 1 |
| 2+ injuries | 640 | 1.45 (1.33-1.57) | 444 | 1.43 (1.30-1.58) | 196 | 1.47 (1.27-1.70) |
| Full siblings/ | 12453 |  | 8926 |  | 3527 |  |
| No injuries | 10209 | 1 | 7291 | 1 | 2918 | 1 |
| >=1 injury | 2244 | 1.39 (1.33-1.46) | 1635 | 1.42 (1.34-1.5) | 609 | 1.33 (1.22-1.46) |
| 0 | 10209 | 1 | 7291 | 1 | 2918 | 1 |
| ]0-1] | 1691 | 1.33 (1.27-1.41) | 1213 | 1.33 (1.25-1.42) | 478 | 1.34 (1.22-1.48) |
| ]1-2] | 411 | 1.54 (1.39-1.70) | 321 | 1.71 (1.52-1.91) | 90 | 1.14 (0.93-1.41) |
| >2 | 142 | 1.81 (1.54-2.14) | 101 | 1.80 (1.48-2.19) | 41 | 1.85 (1.36-2.52) |
| Maternal half siblings | 3486 |  | 2366 |  | 1120 |  |
| No injuries | 2633 | 1 | 1779 | 1 | 854 | 1 |
| >=1 injury | 853 | 1.28 (1.18-1.4) | 587 | 1.32 (1.20-1.46) | 266 | 1.21 (1.04-1.39) |
| Paternal half siblings | 3041 |  | 2102 |  | 939 |  |
| No injuries | 2321 | 1 | 1616 | 1 | 705 | 1 |
| >=1 injury | 720 | 1.18 (1.08-1.29) | 486 | 1.15 (1.04-1.28) | 234 | 1.23 (1.06-1.43) |

^a^ Estimates were adjusted for sex and birth year of the child, as well as the interaction between sex and birth year. Estimates of parental

injury exposures were additionally adjusted for the parent’s birth year. Estimates of sibling injuries were additionally adjusted for the

number of siblings (full, maternal – and paternal half siblings, respectively).

# Abbreviations: ADHD: attention deficit hyperactivity disorder, HR: Hazard ratio.

#

# References

1. Vilhjalmsson BJ, Yang J, Finucane HK, et al. Modeling Linkage Disequilibrium Increases Accuracy of Polygenic Risk Scores. Am J Hum Genet 2015 Oct 1;97(4):576-592.

2. Cross-Disorder Group of the Psychiatric Genomics C, Lee SH, Ripke S, et al. Genetic relationship between five psychiatric disorders estimated from genome-wide SNPs. Nat Genet 2013 Sep;45(9):984-994.

3. Loh PR, Tucker G, Bulik-Sullivan BK, et al. Efficient Bayesian mixed-model analysis increases association power in large cohorts. Nat Genet 2015 Mar;47(3):284-290.

4. Pedersen CB, Bybjerg-Grauholm J, Pedersen MG, et al. The iPSYCH2012 case-cohort sample: new directions for unravelling genetic and environmental architectures of severe mental disorders. Mol Psychiatry 2018 Jan;23(1):6-14.

5. Albinana C, Grove J, McGrath JJ, et al. Leveraging both individual-level genetic data and GWAS summary statistics increases polygenic prediction. Am J Hum Genet 2021 Jun 3;108(6):1001-1011.

6. Loh PR, Bhatia G, Gusev A, et al. Contrasting genetic architectures of schizophrenia and other complex diseases using fast variance-components analysis. Nat Genet 2015 Dec;47(12):1385-1392.

7. Prive F, Luu K, Blum MGB, McGrath JJ, Vilhjalmsson BJ. Efficient toolkit implementing best practices for principal component analysis of population genetic data. Bioinformatics 2020 Aug 15;36(16):4449-4457.

8. Chang CC, Chow CC, Tellier LC, Vattikuti S, Purcell SM, Lee JJ. Second-generation PLINK: rising to the challenge of larger and richer datasets. GigaScience 2015;4:7.

9. Purcell S, Neale B, Todd-Brown K, et al. PLINK: a tool set for whole-genome association and population-based linkage analyses. American Journal of Human Genetics 2007;81(0002-9297; 0002-9297; 3):559-575.

10. Shaun Purcell CC. Plink v1.90b3v 64-bit. v1.90b3v 64-bit ed; 2015.

11. Lee SH, Goddard ME, Wray NR, Visscher PM. A better coefficient of determination for genetic profile analysis. Genet Epidemiol 2012 Apr;36(3):214-224.
